# Evidence that DNA repair genes, a family of tumor suppressor genes, are associated with evolution rate and size of genomes

**DOI:** 10.1186/s40246-019-0210-x

**Published:** 2019-06-07

**Authors:** Konstantinos Voskarides, Harsh Dweep, Charalambos Chrysostomou

**Affiliations:** 1 Medical School, University of Cyprus, Kallipoleos 75, 1678 Nicosia, Cyprus; 2 The Wistar Institute, Philadelphia, PA USA; 3 The Cyprus Institute, Nicosia, Cyprus

**Keywords:** Genomics, Evolutionary genetics, Natural selection, Rapid evolution, Speciation, Mutagenesis rate, Evolutionary medicine, Molecular evolution

## Abstract

Adaptive radiation and evolutionary stasis are characterized by very different evolution rates. The main aim of this study was to investigate if any genes have a special role to a high or low evolution rate. The availability of animal genomes permitted comparison of gene content of genomes of 24 vertebrate species that evolved through adaptive radiation (representing high evolutionary rate) and of 20 vertebrate species that are considered as living fossils (representing a slow evolutionary rate or evolutionary stasis). Mammals, birds, reptiles, and bony fishes were included in the analysis. Pathway analysis was performed for genes found to be specific in adaptive radiation or evolutionary stasis respectively. Pathway analysis revealed that DNA repair and cellular response to DNA damage are important (false discovery rate = 8.35 × 10^−5^; 7.15 × 10^−6^, respectively) for species evolved through adaptive radiation. This was confirmed by further genetic in silico analysis (*p* = 5.30 × 10^−3^). Nucleotide excision repair and base excision repair were the most significant pathways. Additionally, the number of DNA repair genes was found to be linearly related to the genome size and the protein number (proteome) of the 44 animals analyzed (*p* < 1.00 × 10^−4^), this being compatible with Drake’s rule. This is the first study where radiated and living fossil species have been genetically compared. Evidence has been found that cancer-related genes have a special role in radiated species. Linear association of the number of DNA repair genes with the species genome size has also been revealed. These comparative genetics results can support the idea of punctuated equilibrium evolution.

**Electronic supplementary material:**

The online version of this article (10.1186/s40246-019-0210-x) contains supplementary material, which is available to authorized users.

## Background

Adaptive radiation is a well-known phenomenon in evolutionary biology, where a taxon is split in multiple species which become adapted in a variety of environments in short evolutionary time. Although this phenomenon is mostly known in islands like the great examples of Darwin finches [1] and the Hawaiian drosophilas, other major adaptive radiations have occurred in other animals like cichlids, bats, and cetaceans [2–5]. It is very likely that common evolutionary and molecular processes have been followed in all taxa that have experienced adaptive radiation [6, 7]. No such common molecular pathways have been identified so far.

We could consider living fossil species and adaptive radiation as two very different evolutionary strategies: slow evolutionary rate versus rapid evolutionary rate respectively. Living fossils are characterized by morphological stasis, low taxonomic diversity, and certain rareness. Quantitative criteria have been published recently [8, 9]. The apparent absence of diversification and their morphological stability suggest highly effective adaptations that reduce the need for phenotypic change, regardless of environmental or genetic changes [8, 10]. Living fossils are frequently referred to as an example of evolutionary success and evolutionary stasis [11, 12]. Evolutionary stasis is a common finding in the fossil record [13]. The punctuated equilibrium theory of evolution is based on these fossil observations [14, 15]. Characteristic examples of taxa that are considered by most biologists as living fossils are the crocodilians, coelacanths, and ornithorhynchus. Like in the case of adaptive radiation, our knowledge is insufficient for any special genes that are under selection in living fossil species.

This study was mainly aiming at the identification of any common molecular pathways that contributed to a special evolutionary process in animals. We are mostly interested on genes that are related with disease, since evolutionary studies may contribute to a better understanding of the function of those genes. We supposed that living fossil species (LF) and radiated species (R, those that have been evolved through adaptive radiation) represent two animal categories with a very different rate and form of evolution. We took advantage of the plentiful animal genomes that have been sequenced since presently, and we performed an analytical comparative genetics study. Strict inclusion and statistical criteria were applied (see the “Methods” section). In total, 20 LF and 24 R vertebrate genomes (bony fishes, reptiles, birds, mammals) have been analyzed. Interestingly, only one major genetic difference was revealed related to DNA repair genes, one of the most important categories of tumor suppressor genes.

## Methods

### Species included in this study—genome data

The literature was carefully searched for all animal species that can be characterized as living fossils (LF) (slow evolutionary rate) or radiated (R) (they have experienced adaptive radiation). Additional inclusion criteria are as follows: species with a completed genome project, species with available annotation and gene symbol data (for reliable interspecies comparison). Annotation of genomes has been performed by the submitters under the same NCBI standards. We included animal classes with representative species in both living fossil species and radiated species for a reliable comparison. Genome and gene data used for this work are updated since April of 2019, according to Genome and Gene databases of NCBI (https://www.ncbi.nlm.nih.gov/). In total, 44 species were included in this analysis.

### Gene analysis

Official gene symbols were used for comparison among species. A custom algorithm was developed for finding all common genes in the LF species group and in the R species group. Next, the two lists of common genes were compared. This was performed through the “unique values” function of Excel 2016. After comparison, two gene lists were created: genes that are common in LF but not found in R and genes that are common in R and not found in LF. We considered that these genes are probably associated with a special type of evolutionary process. Genes were analyzed under the concept of presence/absence. Copy numbers were not considered. All gene lists can be found in Additional file 1: Table S1.

### Pathway analysis and DNA repair gene analysis

Panther 14.1 online software [16, 17] was used for pathway analysis of the two LF and R unique gene lists. The software analyzes the submitted gene lists with reference to the human genome. Two algorithms of the software were used: pathway and reactome profile analysis. Results were compared between LF and R to find any pathways that are unique in any of the two evolutionary processes. False discovery rate (FDR) is the statistical outcome that is a special type of adjusted *p* value. Significant level alpha was set to 0.0001 for highly reliable results.

To confirm if DNA repair genes represent a major genetic difference between the two vertebrate categories, all 44 species’ genomes were analyzed for their content in DNA repair genes. An updated list of all 151 known DNA repair genes was used [18]. Content analysis (presence/absence) was performed using the official gene symbols. An extra search was performed using the gene aliases for any missed misnamed genes. Content analysis was performed through the “duplicate values” function of Excel 2016. Results in detail can be found in Additional file 2: Table S2.

### Statistical analysis

All statistical analysis needed for this work was performed through the statistical package STATAv.13 (StataCorp LLC, Texas, USA). The basic statistical analysis included univariate linear regression and independent *t* test (two-tailed). The heat map was performed through the “color gradient” function of Excel 2016. Significant level alpha was set to 0.01 for identifying the most significant categories of DNA repair genes.

## Results and discussion

### Species analyzed

Strict inclusion criteria were applied for the 44 species analyzed in this study. Several fossil and molecular studies that are cited below justify the classification “living fossil” or “radiated.” A more detailed description of “living fossil” species can be found in the book *Living Fossils* of [19]. Additionally, the 20 LF species satisfy the very accurate living fossil quantification system of [9]. Genome projects information can be found in Table 1.

**Table 1:** Living fossil (LF) vertebrate species and radiated (R) vertebrate species analyzed in this study, with genome and proteome information

Species	Genome size (Mb)	Protein number	Genome projects
Mammals—LF			
L1. *Orycteropus afer*	4566	25,544	https://www.ncbi.nlm.nih.gov/genome/annotation_euk/
L2. *Ornithorhynchus anatinus*	1924	24,786	[22, 73]
L3. *Monodelphis domestica*	3598	49,112	[74]
L4. *Elephantulus edwardii*	4066	25,209	https://www.ncbi.nlm.nih.gov/genome/annotation_euk/
L5. *Ailuropoda melanoleuca*	2364	36,506	[75, 76]
L6. *Phascolarctos cinereus*	3398	46,908	[77]
L7. *Carlito syrichta*	3454	33,081	[78]
Mammals—R			
R1. *Myotis brandtii*	2107	40,808	[79]
R2. *Pteropus alecto*	1986	39,227	[80]
R3. *Rousettus aegyptiacus*	1941	48,803	[81]
R4. *Myotis davidii*	2060	33,106	[80]
R5. *Hipposideros armiger*	2237	45,831	[82]
R6. *Myotis lucifugus*	2035	43,106	[83]
R7. *Pteropus vampyrus*	2198	43,628	https://www.ncbi.nlm.nih.gov/genome/annotation_euk/
R8. *Miniopterus natalensis*	1803	29,787	[84]
R9. *Eptesicus fuscus*	2027	49,822	https://www.ncbi.nlm.nih.gov/genome/annotation_euk/
R10. *Microcebus murinus*	2487	59,023	https://www.ncbi.nlm.nih.gov/genome/annotation_euk/
R11. *Propithecus coquereli*	2798	28,194	https://www.ncbi.nlm.nih.gov/genome/annotation_euk/
R12. *Tursiops truncatus*	2478	38,849	[85, 86]
R13. *Balaenoptera acutorostrata*	2432	37,625	[87, 88]
R14. *Physeter catodon*	2512	50,591	https://www.ncbi.nlm.nih.gov/genome/annotation_euk/
R15. *Orcinus orca*	2373	27,870	[85, 89, 90]
R16. *Lipotes vexillifer*	2429	26,901	[91]
R17. *Delphinapterus leucas*	2358	49,714	[92]
Birds and reptiles—LF			
L8. *Pelecanus crispus*	1161	16,298	[93]
L9. *Acanthisitta chloris*	1036	16,077	[93]
L10. *Colius striatus*	1076	15,797	[93]
L11. *Cariama cristata*	1132	16,125	[93]
L12. *Tinamus guttatus*	1047	17,873	[93]
L13. *Opisthocomus hoazin*	1203	14,878	[93]
L14. *Crocodylus porosus*	2085	28,676	[38, 94]
L15. *Alligator mississippiensis*	2162	42,388	[38, 94]
L16. *Gavialis gangeticus*	2415	27,294	[38, 94]
L17. *Alligator sinensis*	2271	43,105	[95]
Birds and reptiles—R			
R18. *Geospiza fortis*	1065	16,724	[67, 93, 96]
R19. *Parus major*	1020	39,666	[97, 98]
R20. *Anolis carolinensis*	1799	34,827	[69]
Bony fishes—LF			
L18. *Scleropages formosus*	742	32,859	[39, 99, 100]
L19. *Lepisosteus oculatus*	946	41,647	[40]
L20. *Latimeria chalumnae*	2798	34,251	[101, 102]
Bony fishes—R			
R21. *Notothenia coriiceps*	637	31,979	[103]
R22. *Maylandia zebra*	957	46,173	[59, 104]
R23. *Pundamilia nyererei*	830	38,583	[59, 105]
R24. *Haplochromis burtoni*	831	44,653	[59, 105, 106]

The 20 LF species or taxa are as follows (common names, scientific names are found in Table 1): aardvark [20], platypus [21, 22], opossum [23, 24], elephant shrew [25], giant panda [26], koala [23, 27], Philippine tarsier [28], pelican [29], New Zealand wren [30, 31], speckled mousebird [32], red-legged seriema [33], tinamou [34], hoatzin [35–37], crocodilians [38], arowana [39], spotted gar [40], and coelacanth [12, 41].

The 24 R species or taxa are as follows (common names, scientific names are found in Table 1): bats [42–44], dolphins and whales [45, 46], lemurs [47–49], medium ground finch [50, 51], great tit [51], Carolina anole [52–55], black rockcod [56–58], and three cichlid species [59–62].

### Gene and pathway analysis

Evolutionary stasis and rapid evolutionary speciation can be characterized as opposite evolutionary procedures or at least very different evolutionary phenomena. This is the first study that compares genetically those two very different categories of vertebrate species. Gene or annotation information was inadequate for most invertebrate LF or R species, so they were not included in this study.

The procedure we followed is very simple. We downloaded the annotated genome information for all 44 species. Then, we found the common genes in LF species and the common genes in R species, creating two separate gene lists (Additional file 1: Table S1). The next step was to compare the two lists to find any genes that are common in LF but not found in R species and genes that are common in R but not found in LF species. We consider that these genes may be under selection since they are found only in species with a special evolutionary profile. In total, 1534 genes were found to be specific for LF species and 2263 genes to be specific for R species.

Analysis of the two final gene lists was performed by Panther 14.1 software, under two algorithms: pathways (biological processes) and reactome. We looked for unique biological processes and reactomes in LF- and R-specific genes respectively. Using the strict criterion of FDR ≤ 0.0001, only one process/pathway was found to be significant in R-specific genes by both algorithms, this being DNA repair (DNA repair and cellular response to DNA damage; FDR = 8.35 × 10^−5^ and 7.15 × 10^−6^, respectively). Not any common significant pathways came out in the biological processes and reactome analyses for LF-specific genes. Step by step analysis and all analytical output can be found in Additional file 1: Table S1. The flowchart of analysis can be found in Table 2.

**Table 2:** Flowchart and main outcomes of each analysis performed in this study

Analysis	Outcome
Identification of all vertebrate species that can be characterized as living fossil or radiated species, with available whole genome sequencing data and complete gene annotation	20 living fossil species 24 radiated species
Genes in common per group	Living fossil species: 2861 genes in common Radiated species: 3590 genes in common
Genes in common per group, not found in the other group	Living fossil species: 1534 unique genes Radiated species: 2263 unique genes
Pathway (biological processes) and reactome analyses, unique ones	Living fossil species: 0 pathways, 2 reactomes Radiated species: 7 pathways, 2 reactomes
Significant process revealed by both algorithms	Living fossil species: None Radiated species: DNA repair and cellular response to DNA damage (FDR = 8.35 × 10^−5^; 7.15 × 10^−6^, respectively)
Search for 151 known DNA repair genes in the 45 species’ genomes Mean comparison analysis	More DNA repair genes in radiated species than in living fossil species (*p* = 5.3 × 10^−3^) Most significant gene subcategory: Nucleotide excision repair (*p* = 5.00 × 10^−4^)
Linear regression: DNA repair genes number vs genome size or protein number	Genome size/protein number is linearly related with the number of DNA repair genes (*p* < 1.0 × 10^−4^)

*FDR* false discovery rate

### DNA repair gene analysis

**Fig. 1:**
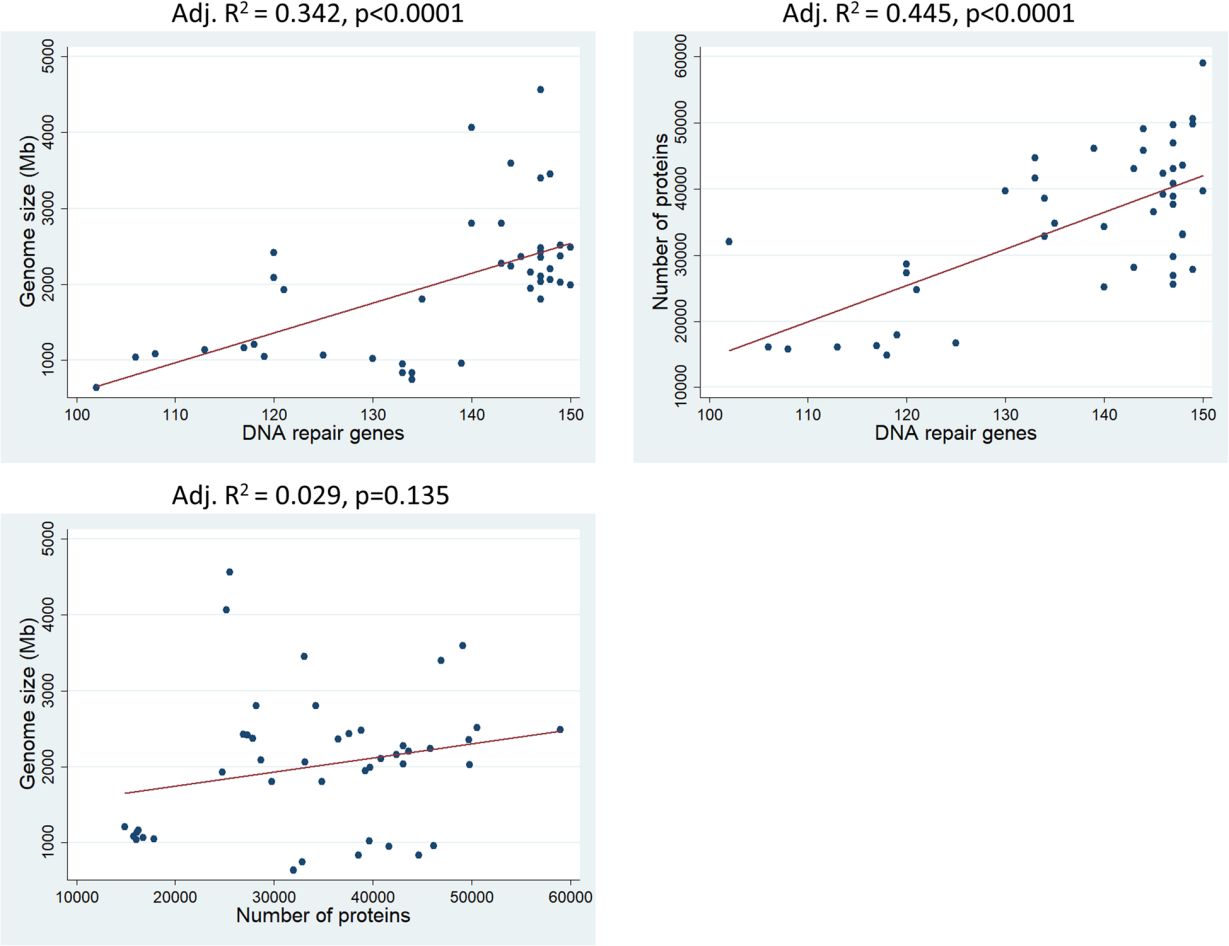
Heat map showing the quantity of DNA repair genes, from red to blue in ascending order, per species’ genome (numbers at the top of the figure represent the species code that is found in Table 1). Each DNA repair gene pathway was analyzed separately in rows. Radiated species’ genomes are richer in DNA repair genes. Analytical data can be found in Additional file 2: Table S2. M mammals, B&R birds and reptiles, BF bony fishes

In order to confirm the pathway analysis results, we analyzed the 44 genomes for their content in DNA repair genes, using a list of all known DNA repair genes since presently (updated list of Wood et al. [18]). Subcategories of DNA repair genes were also considered in the analysis. Results in detail can be found in Additional file 2: Table S2. The results highly confirmed the previously performed pathway analysis (Table 3). R species’ genomes are significantly enriched in DNA repair genes (*p* = 5.3 × 10^−3^). The most significant subcategories are the nucleotide excision repair (*p* = 5.00 × 10^−4^) and base excision repair (*p* = 9.80 × 10^−3^). Many other subcategories seem to be significantly enriched in R species under the criterion of *p* < 0.05. Conserved DNA damage response and non-homologousend-joining are not significant at all (Table 3). A heat map diagram shows that indeed the R species’ genomes are enriched in DNA repair genes in comparison with the LF species, especially for mammals, reptiles, and birds (Fig. 1).

**Table 3:** Mean comparison (independent *t* test, two-tailed) between living fossil (LF) and radiated species (R), for each category of DNA repair genes and altogether (degrees of freedom, 42)

DNA repair gene category	Species group	Mean number	Std. dev.	[95% conf. interval]	*t* value	*p* value
Base excision repair (BER)	20 (LF)	16.75	3.274704	15.21739, 18.28261	− 2.7067	*9.80 × 10* ^*−3*^
	24 (R)	18.92	1.976309	18.08214, 19.75119		
Conserved DNA damage response	20 (LF)	15.2	2.876401	13.8538, 16.5462	− 2.2001	3.34 × 10^−2^
	24 (R)	16.83	2.03591	15.97364, 17.69302		
Direct reversal of damage	20 (LF)	2.9	0.3077935	2.755948, 3.044052	− 0.1872	0.8524
	24 (R)	2.92	0.2823299	2.797449, 3.035884		
DNA polymerases	20 (LF)	14.3	1.688974	13.50954, 15.09046	− 2.4279	1.96 × 10^−2^
	24 (R)	15.38	1.244553	14.84947, 15.90053		
Editing and processing nucleases	20 (LF)	6.5	1.100239	5.985072, 7.014928	− 2.4341	1.93 × 10^−2^
	24 (R)	7.25	0.9440892	6.851346, 7.648654		
Fanconi anemia	20 (LF)	13.55	0.9445132	13.10795, 13.99205	− 2.2591	2.91 × 10^−2^
	24 (R)	14.21	0.9770927	13.79574, 14.62092		
Homologous recombination	20 (LF)	21.2	2.261811	20.14144, 22.25856	− 1.6880	9.88 × 10^−2^
	24 (R)	22.33	2.180281	21.41268, 23.25399		
Mismatch excision repair (MMR)	20 (LF)	8.35	0.8127277	7.969632, 8.730368	− 1.7706	8.39 × 10^−2^
	24 (R)	8.79	0.8329709	8.439934, 9.143399		
Non-homologous end-joining	20 (LF)	6.55	0.6863327	6.228786, 6.871214	− 0.8497	0.4003
	24 (R)	6.71	0.5500329	6.476075, 6.940592		
Nucleotide excision repair (NER)	20 (LF)	25.15	3.528456	23.49863, 26.80137	− 3.8043	*5.00 × 10* ^*−4*^
	24 (R)	28.46	2.186503	27.53505, 29.38161		
All DNA repair genes	20 (LF)	130.45	14.56916	123.6314, 137.2686	−2.9417	*5.30 × 10* ^*−3*^
	24 (R)	141.79	10.99003	137.151, 146.4324

The top 20 genes with the highest existence rate in R species in relation to LF species can be found in Additional file 2: Table S2. Eleven out of the top 20 (55%) are genes related with nucleotide excision repair and base excision repair. All gene rates are available in Additional file 2: Table S2.

### Genome and proteome size analysis

**Fig. 2:**
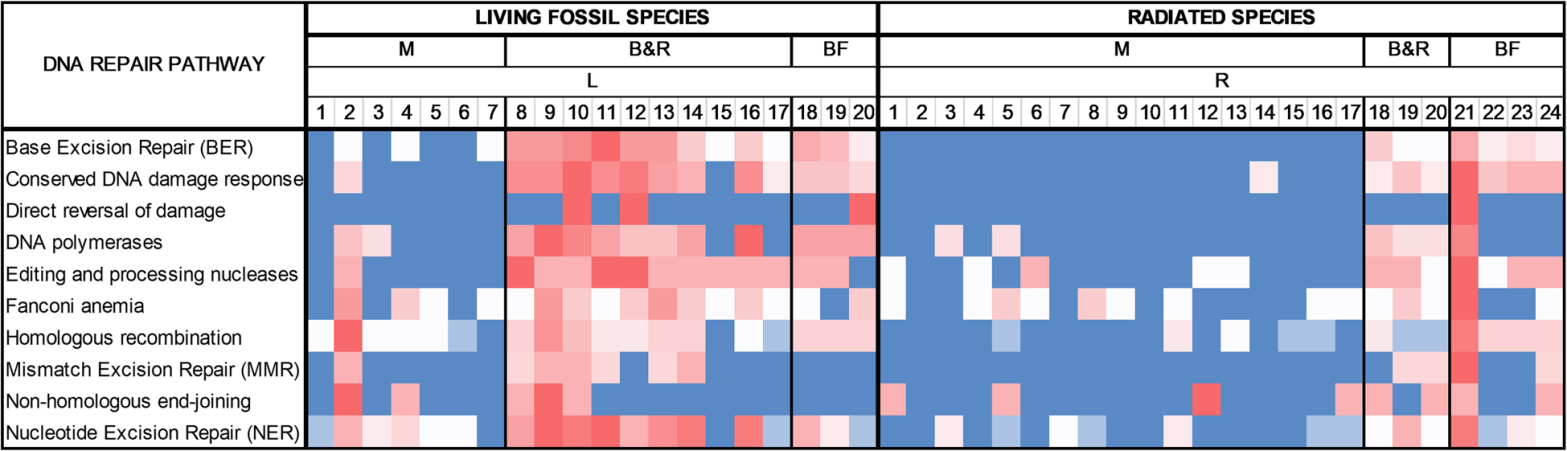
Linear regression analysis. The number of DNA repair genes is linearly related to genome size and protein number. As a negative control, we show that genome size is not linearly related with protein number

Interestingly, the number of DNA repair genes is linearly related with the genome size and the number of proteins (*p* < 1.00 × 10^−4^). We used genome and proteome data (https://www.ncbi.nlm.nih.gov/) of the 44 vertebrate species (Fig. 2). The two linear associations are independently significant since genome size is not linearly related with the number of proteins (Fig. 2). It is well known that genome size is not related with organism complexity [63]; thus, we consider that this association is not due to increased complexity of large genomes. Not any association was found when genome size means of LF and R species were compared (results not shown).

This result may also explain Drake’s rule. This is about the density of accumulated mutations per generation (mutagenesis rate) that is roughly inversely proportional to genome size [64–66]. Here, we found that larger genomes have more DNA repair genes (and possibly lower mutagenesis rate, if DNA errors are corrected at a higher rate) that may explain Drake’s rule, being unexplained for years.

### Why DNA repair genes

There is evidence that LF species are evolving slower than R species. Additionally, some data show that mutagenesis and nucleotide diversity [59, 67] may be higher in R species than in LF species and that some R species with huge bodies (whales) have duplicated DNA repair genes to be protected by cancer [68, 69]. According to these data, we could hypothesize that R species may be at risk due to high mutation load. This could be balanced with more DNA repair genes, repairing as much DNA damages as possible. It seems that DNA repair at the nucleotide level (nucleotide excision repair and base excision repair) is more important than other DNA repair pathways (Table 3, Additional file 2: Table S2). Another explanation is that LF species are probably more protected from spontaneous DNA changes since due to the vast evolutionary time that they exist, stabilizing selection has formed their genome in a way that they are protected from random DNA changes that could change their general morphological features. Certain genes in LF genomes may act in a canalizing way that keeps these species in a narrow state of development and evolution since they are evolutionary successful. R species are not characterized by those features, and probably they need more or certain DNA repair genes to continue to diversify under a non-deleterious mutagenesis rate. We could consider that this is the first evidence for genes related with punctuated equilibrium evolution (long evolutionary stasis followed by short speciation explosions) [14, 15].

The fact that the number of DNA repair genes is related with the genome and proteome size is quite logical since larger genomes need more protection from spontaneous mutagenesis. This is the first time that a class of genes has been associated with genome size and number of proteins in animals.

## Conclusions

A big number of genomes have been compared under the prism of evolutionary stasis and adaptive radiation. The analysis concluded that DNA repair genes might play a previously unknown significant role in evolution. It seems that more DNA repair genes are found in vertebrate taxa that have experienced recent adaptive radiation. Additionally, DNA repair genes were found to be statistically associated with the genome size and protein number in vertebrates. DNA repair genes are considered as tumor suppressor genes. There is evidence that tumor suppressor genes are related to environmental adaptation in humans [70, 71] and selective pressures along the evolution of mammals [72]. We can imagine that certain evolutionary procedures may be DNA repair-dependent, this showing the way for future analyses and experiments.

## Additional files

Additional file 1: **Table S1.**Pathway analysis by PANTHER (XLSX 137 kb)

Additional file 2: **Table S2.** DNA repair gene analysis (XLSX 95 kb)

## Data Availability

All data generated or analyzed during this study are included in this published article [and its supplementary information files].
