# Correction to: Evidence that DNA repair genes, a family of tumor suppressor genes, are associated with evolution rate and size of genomes

**DOI:** 10.1186/s40246-019-0214-6

**Published:** 2019-07-02

**Authors:** Konstantinos Voskarides, Harsh Dweep, Charalambos Chrysostomou

**Affiliations:** 1 Medical School, University of Cyprus, Kallipoleos 75, 1678 Nicosia, Cyprus; 2 The Wistar Institute, Philadelphia, PA USA; 3 The Cyprus Institute, Nicosia, Cyprus

**Correction to: Hum Genomics (2019) 13:26**

**https://doi.org/10.1186/s40246-019-0210-x**

In the original publication of this article [1], the Fig. 1 and Fig. 2 were wrong. The Fig. 1 “*Heat map showing the quantity of DNA repair genes, from red to blue in ascending order, per species’ genome (numbers at the top of the figure represent the species code that is found in Table 1). Each DNA repair gene pathway was analyzed separately in rows. Radiated species’ genomes are richer in DNA repair genes. Analytical data can be found in Additional file 2: Table S2. M mammals, B&R birds and reptiles, BF bony fishes*” should be the picture of Fig. 2. The Fig. 2 “*Linear regression analysis. The number of DNA repair genes is linearly related to genome size and protein number. As a negative control, we show that genome size is not linearly related with protein number*” should be the picture of Fig. 1.

The correct Figures should be:

**Fig. 1:**
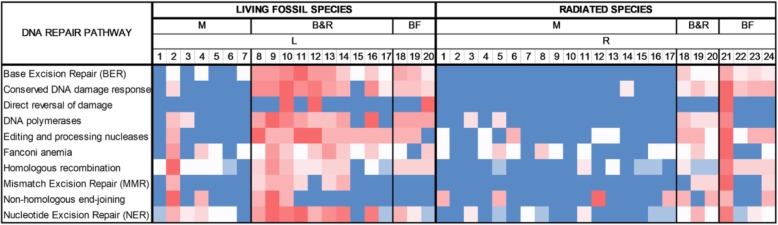
Heat map showing the quantity of DNA repair genes, from red to blue in ascending order, per species’ genome (numbers at the top of the figure represent the species code that is found in Table 1). Each DNA repair gene pathway was analyzed separately in rows. Radiated species’ genomes are richer in DNA repair genes. Analytical data can be found in Additional file 2: Table S2. M mammals, B&R birds and reptiles, BF bony fishes

**Fig. 2:**
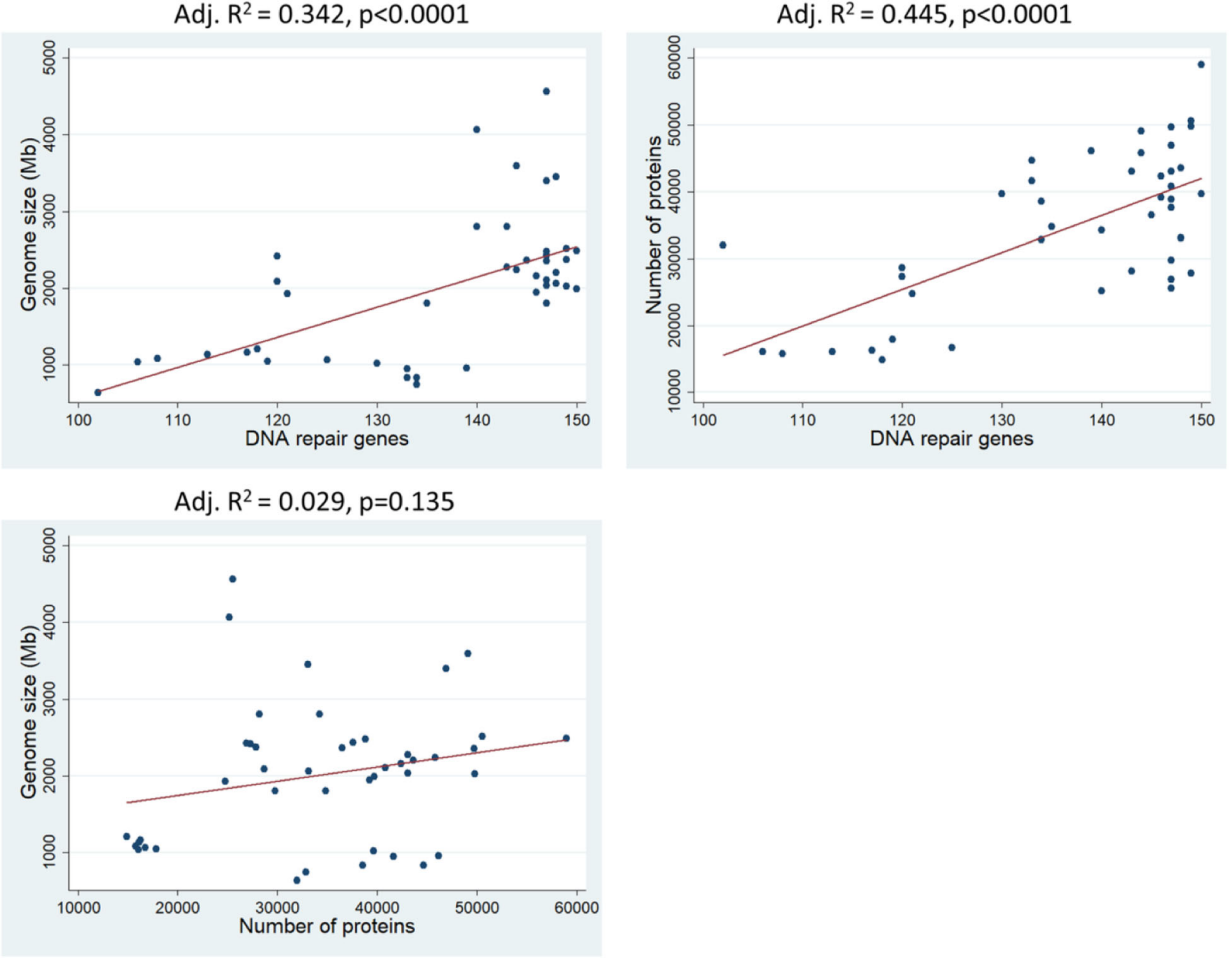
Linear regression analysis. The number of DNA repair genes is linearly related to genome size and protein number. As a negative control, we show that genome size is not linearly related with protein number

The original article has been corrected.
